# Myocardial Infarction-induced N-terminal Fragment of Cardiac Myosin-binding Protein C (cMyBP-C) Impairs Myofilament Function in Human Myocardium*

**DOI:** 10.1074/jbc.M113.541128

**Published:** 2014-02-07

**Authors:** Namthip Witayavanitkul, Younss Ait Mou, Diederik W. D. Kuster, Ramzi J. Khairallah, Jason Sarkey, Suresh Govindan, Xin Chen, Ying Ge, Sudarsan Rajan, David F. Wieczorek, Thomas Irving, Margaret V. Westfall, Pieter P. de Tombe, Sakthivel Sadayappan

**Affiliations:** From the ‡ Department of Cell and Molecular Physiology, Health Sciences Division, Loyola University Chicago, Maywood, Illinois 60153,; the § Human Proteomics Program, School of Medicine and Public Health, University of Wisconsin-Madison, Madison, Wisconsin 53706,; the ¶ Department of Molecular Genetics, Biochemistry, and Microbiology, University of Cincinnati College of Medicine, Cincinnati, Ohio 45267,; the ‖ Department of Biological and Chemical Sciences, Illinois Institute of Technology, Chicago, Illinois 60616, and; the ** Department of Cardiac Surgery, University of Michigan, Ann Arbor, Michigan 48109

**Keywords:** Contractile Protein, Heart Failure, Myocardial Infarction, Protein Degradation, Protein-Protein Interactions

## Abstract

Myocardial infarction (MI) is associated with depressed cardiac contractile function and progression to heart failure. Cardiac myosin-binding protein C, a cardiac-specific myofilament protein, is proteolyzed post-MI in humans, which results in an N-terminal fragment, C0-C1f. The presence of C0-C1f in cultured cardiomyocytes results in decreased Ca^2+^ transients and cell shortening, abnormalities sufficient for the induction of heart failure in a mouse model. However, the underlying mechanisms remain unclear. Here, we investigate the association between C0-C1f and altered contractility in human cardiac myofilaments *in vitro*. To accomplish this, we generated recombinant human C0-C1f (hC0C1f) and incorporated it into permeabilized human left ventricular myocardium. Mechanical properties were studied at short (2 μm) and long (2.3 μm) sarcomere length (SL). Our data demonstrate that the presence of hC0C1f in the sarcomere had the greatest effect at short, but not long, SL, decreasing maximal force and myofilament Ca^2+^ sensitivity. Moreover, hC0C1f led to increased cooperative activation, cross-bridge cycling kinetics, and tension cost, with greater effects at short SL. We further established that the effects of hC0C1f occur through direct interaction with actin and α-tropomyosin. Our data demonstrate that the presence of hC0C1f in the sarcomere is sufficient to induce depressed myofilament function and Ca^2+^ sensitivity in otherwise healthy human donor myocardium. Decreased cardiac function post-MI may result, in part, from the ability of hC0C1f to bind actin and α-tropomyosin, suggesting that cleaved C0-C1f could act as a poison polypeptide and disrupt the interaction of native cardiac myosin-binding protein C with the thin filament.

## Teaser

**Background:** Myocardial infarction (MI) leads to proteolytic cleavage of cMyBP-C (hC0C1f) and decreased contractility.

**Results:** hC0C1f can incorporate into the human cardiac sarcomere, depressing force generation and increasing tension cost.

**Conclusion:** Interaction between hC0C1f and both actin and α-tropomyosin causes disruption of intact cMyBP-C function.

**Significance:** Proteolytic cleavage of cMyBP-C is sufficient to cause contractile dysfunction following MI.

## Introduction

Myocardial infarction (MI)^3^ leads to depressed cardiac contractile function and often progresses to heart failure. This progression is preceded by impaired Ca^2+^ handling, altered myofilament phosphorylation, and reduced cross-bridge cycling rates. Surprisingly, cardiomyocytes in remote noninfarcted regions, unaffected by ischemia, also display decreased contractility, despite restoration of blood flow. The mechanisms leading to this dysfunction are still incompletely understood (1). We have previously shown that MI is associated with catalytic cleavage of cardiac myosin binding protein C (cMyBP-C), an important regulator of cardiac contractility (2).

Following MI, this proteolytic fragment can be detected in the ischemic region but is also present in the noninfarcted adjacent and remote regions, as well as in nonischemic heart failure (2–4), presumably after *in situ* cleavage. Furthermore, cleaved cMyBP-C is released into the blood following MI, such that its N-terminal fragment (C0-C1f) can be used as a marker of MI (2). The C0-C1f fragment has been shown to disrupt myosin-regulated contraction (3) and potentially lead to global dysfunction in the heart, not just in the ischemic area. Therefore, the study aimed to identify the mechanisms underlying altered myosin function by the presence of C0-C1f fragments in the sarcomere, including its interaction with thick and thin filament proteins and its effects on ATPase activity, myofilament Ca^2+^ sensitivity, and contractility.

The C0-C1f fragment contains the cardiac-specific C0 domain, the proline-alanine (Pro-Ala)-rich region, the C1 domain, and the first 17 residues of the M domain (1–271 residues; C0-C1f) of cMyBP-C (2). The N-terminal region of cMyBP-C interacts with both actin and myosin and is believed to function as a critical regulator of contraction (5–7). The cardiac isoform of cMyBP-C differs from the two skeletal isoforms by having an extra C0 domain at the N′-end (8). Thus, the C0 domain is exclusively unique and specific to cMyBP-C. The C0 domain is able to directly bind with both actin (9, 10) and regulatory light chain of myosin (11), thereby placing the N-terminal region of cMyBP-C in close proximity to the motor domain of myosin (11). However, the relative binding affinity of the C0 domain to actin was found to be less than that of C1-C2 (9, 10). The C1-M-C2 region binds to the S2 fragment of myosin and directly influences myofilament Ca^2+^ sensitivity, myofibrillar tension development, cross-bridge cycling kinetics, and sarcomere length (SL)-tension relationships (5–7). The C1-M-C2 region also binds to actin, supporting the idea that cMyBP-C may play a role in the regulation of cardiac contraction by modulating actin-myosin interaction (9, 10, 12, 13). Furthermore, the interaction of the C1-M-C2 region of cMyBP-C with actin and myosin is dynamically regulated in an on-off fashion by phosphorylation within the M domain (6, 9, 13, 14). Phosphorylation by PKA prevents the cMyBP-C N′-region from binding to actin (9) and myosin S2 (15) or altering myofilament Ca^2+^ sensitivity (16). Interestingly, the cleaved C0-C1f fragment generated by the proteolytic degradation of cMyBP-C following ischemia/reperfusion (I/R) injury does not contain these regulatory phosphorylation sites (2). Because the C0-C1f region retains strong interaction with actin but lacks the phosphorylation sites necessary for phosphorylation-dependent on-off interaction with myosin and actin, we hypothesized that the presence of the C0-C1f fragments in the sarcomere would alter actin-myosin interaction by constitutively interacting with thin filament proteins, such as actin and α-tropomyosin (α-TM), in turn having direct consequences on force generation of sarcomere function.

We have previously shown that expression of C0-C1f protein in neonatal and adult cardiomyocytes induces contractile dysfunction (4). Furthermore, transgenic mice expressing this fragment display sarcomere dysgenesis, increased fibrosis, and impaired contractility, leading to the development of heart failure (3), abnormalities suggesting that cleaved C0-C1f could act as a poison polypeptide. We hypothesize that C0-C1f interferes with the binding of native cMyBP-C to thick and thin filaments, thereby altering its regulation of actomyosin interaction. This would then result in the depressed myocardial function observed in post-MI and in heart failure. Here, to determine the impact of C0-C1f on sarcomere function, we applied human C0-C1f (hC0C1f) recombinant protein fragment to permeabilized donor human myocardium and assayed force-Ca^2+^/force-ATPase relationships, length-dependent activation, and cross-bridge cycling kinetics. We show that hC0C1f increases cross-bridge cycling kinetics and tension cost, effectively breaking the interaction between cMyBP-C and actomyosin. Further, we establish that the effects of hC0C1f occur through direct interaction with the thin filament proteins actin and α-TM.

## EXPERIMENTAL PROCEDURES

### 

#### 

##### Human Samples

Non-age/sex-matched, deidentified hearts were procured via the National Disease Research Interchange. Tissue procurement and processing was approved by the University of Michigan Institutional Review Board, and the Institutional Review Board at Loyola University Chicago approved the protocol for the use of deidentified human donor hearts. Prior to explant, hearts were flushed with ice-cold cardioplegia solution and arrived on ice <12 h postexplant. Sections of left ventricular tissue were then immediately frozen in liquid N_2_ and stored at −80 °C until use.

##### Top-Down Analysis by Mass Spectrometry

His-tagged recombinant hC0C1f proteins were expressed in *Escherichia coli* and purified as described previously (4, 15). Recombinant hC0C1f (20–100 μg) was desalted by offline reverse phase protein trap (Michrom Bioresources). The samples were introduced to the mass spectrometer via an automated chip-based nano-electrospray ionization source (Triversa NanoMate; Advion BioSciences, Ithaca, NY) with a spray voltage of 1.2–1.6 kV *versus* the inlet of the mass spectrometer, resulting in a flow of 50–200 nl/min. Intact protein molecular ions were analyzed using 7T linear ion trap/FTICR (LTQ FT Ultra) hybrid MS (Thermo Scientific Inc., Bremen, Germany) as described previously (17). Up to 1,500 transients were averaged per spectrum to ensure high quality spectra. All FTICR spectra were processed with in-house developed MASH Suite software (version 1.0), using the THRASH algorithm with a signal to noise threshold of 3 and fit factor of 60% and validated manually.

##### Single Permeabilized Cardiac Myocyte Preparation

Single skinned cells were prepared as previously described (18, 19) with adaptations to allow the use of human tissue. Briefly, frozen human left ventricle samples were thawed in ice-cold relaxing solution containing 10 mm EGTA, 10 mm creatine phosphate, 10 units/ml creatine kinase, 100 mm *N,N*-bis(2-hydroxyethyl)taurine; *N,N*-Bis-(2-hydroxyethyl)-2-aminoethanesulfonic acid, 6.3 mm ATP, 6.48 mm magnesium chloride, 49.76 mm potassium propionate, and protease inhibitors mixture (Sigma-Aldrich) at pH 7.0. Single cells were obtained by mechanically homogenizing left ventricular tissues for 3 s at 10,000 rpm using a homogenizer (PowerGen 700D; Fisher Scientific). The homogenate was centrifuged at 120 × *g* for 1 min, and the resulting pellet was filtered to remove any unhomogenized tissue. The run-through was washed with fresh ice-cold relaxing solution and resuspended in relaxing solution containing 1% Triton X-100 for 30 min at room temperature under agitation to permeabilize the cells. The resulting skinned cells were washed three times with ice-cold relaxing solution and suspended in 1.5 ml of fresh ice-cold relaxing solution. The cells were kept on ice and used the same day.

##### 5-Iodoacetamidofluorescein Labeling of hC0C1f

To determine whether the hC0C1f correctly localized to the sarcomere after incubation, hC0C1f was labeled with a thiol-sensitive maleimide fluorophore (5-iodoacetamidofluorescein (IAF)). The labeling procedure was conducted according to the protocol provided by Molecular Probes. Briefly, labeled hC0C1f-IAF was prepared by dissolving lyophilized hC0C1f in dissolving buffer containing 6 m urea, 0.02 m Tris-HCl, and 0.001 m DTT at pH 8.0 to an initial concentration of ∼2 mg/ml and incubated at room temperature until completely dissolved (∼3 h). The DTT was removed by dialyzing the solution against a DTT-free buffer containing 6 m urea, 0.1 m NaCl, 0.02 m Tris-HCl, and 0.001 m EDTA, at pH 7.5, overnight at 4 °C. To ensure high labeling efficiency, hC0C1f was incubated with a 4× molar excess of IAF for 8 h at room temperature. IAF covalently binds via disulfide bonds with cysteine residues. After 8 h, the reaction was stopped by adding 1 mm DTT. To remove unbound IAF, the solution was dialyzed against a postlabeling buffer containing 6 m urea, 1 m NaCl, 0.02 m Tris-HCl, 0.005 m MgCl_2_, and 0.001 m DTT at pH 8.0, overnight at 4 °C. Finally, the solution was dialyzed against relaxing solution overnight at 4 °C. The stock solution was aliquoted (10 μl) and stored at −80 °C. Labeling efficiency was based on the relative molar concentrations of dye/protein, where the dye concentration was determined spectroscopically by absorbance at 492 nm using a molar extinction coefficient of 80,000–85,000 m^−1^ cm^−1^, and the protein concentration was determined by the Bradford method.

##### Localization of C0-C1f after Exchange in Permeabilized Cardiomyocytes

Permeabilized human cardiac myocytes were attached to two needles controlled by a custom micromechanics system using optical glue (NOA 63; Norland Products Inc.) and mounted on a laser scanning confocal microscope (LSM 410; LSM Tech Inc.). After attachment, nonspecific binding was blocked by incubating in blocking solution containing 1% (w/v) BSA, 0.1% (w/v) gelatin, and 0.1% (v/v) Tween 20 for 30 min at room temperature. Mouse monoclonal cardiac α-actinin (Sigma; #A7811) was used to stain the Z-disc at a 1:200 dilution in blocking buffer overnight at room temperature. Unbound antibody was removed by washing three times with PBS. The secondary antibody, goat anti-mouse IgG (Invitrogen; A11001; 1:100 dilution in blocking buffer) conjugated to a fluorescein 488, was then added in blocking solution for 1 h at room temperature. Unbound antibody was washed away with PBS. The cell was then attached and imaged at 488/525 excitation/emission. Following the first image, the cell was then incubated with excess of hC0C1f-IAF (30 μm) for 20 min at room temperature. The unbound hC0C1f-IAF was washed three times with relaxing solution, and the cell was imaged again at 488/525. Both α-actinin and hC0C1f-IAF fluorescence signals were measured simultaneously. The resulting image was then subtracted from the α-actinin staining to reveal the localization of the hC0C1f-IAF. The experiment was also repeated, starting with hC0C1f-IAF incubation and followed by α-actinin staining.

##### X-ray Diffraction

Experiments were performed using the BioCAT undulator-based beamline at the Advanced Photon Source (Argonne National Labs, Lemont, IL) (20). The high x-ray flux density and low beam divergence delivered by this instrument are highly advantageous for small angle x-ray studies of small specimens, such as those described here. Experiments were done using a 3-m distance between the sample and the detector and with the x-ray beam energy set to 12 keV (1.03 Å wavelength). All flight paths were evacuated, except for a small gap around the sample chamber itself (∼1 mm downstream, 2 mm upstream of the sample). The beam size at the sample position was collimated to about 0.4 by 0.8 mm and about 0.065 by 0.15 mm at the detector and contained a maximum incident flux of ∼3 × 10^12^ photons/s. Permeabilized human cardiac tissue was clipped using aluminum T-clips and mounted in a small trough with dimensions of 0.8 mm wide × 40 mm long × 5 mm deep. The bottom of the trough was a glass coverslip allowing the muscle to be viewed by a long working distance (∼4 mm) 4× objective and also allowed for sarcomere length measurement via laser diffraction (21). One end of the trough expanded into a larger reservoir for mounting the fiber. The slides of the trough were hollowed out, allowing the x-ray beam to pass through the fiber via Kapton^TM^ windows 0.0005 inch thick. The aluminum clips holding the fiber were mounted on hooks, slightly larger than the holes in the clips, so that they were held securely between a force transducer (KG7; World Precision Instruments, Sarasota, FL) and a servomotor (Cambridge Technologies model 308B; Aurora Scientific, Aurora, Canada) mounted on micromanipulators. During the experiment, relaxing solution was continuously perfused through the chamber using a peristaltic pump. All experiments were done at room temperature. A highly sensitive CCD detector (Aviex PCCD 16080) with a 160-mm × 80-mm active area and 39-μm pixels was used to collect x-ray patterns. Prior to analysis, x-ray diffraction patterns were corrected for dark current flat field and spatial distortions. Spacings on detector images were measured using the FIT2D program on a UNIX work station. The accuracy of these measurements reached to ±1 pixel of 200–600 (*i.e.*, <0.5%) or better. Measured spacings of the 1,0 and 1,1 equatorial reflections from the diffraction pattern were converted to d_1,0_ lattice spacing (thin filament spacing) using Bragg's Law, which can then be converted to the interfilament spacing.

##### Simultaneous Measurement of Isometric Tension and ATPase Activity

Permeabilized human cardiac tissue was obtained by adapting previously described methods (19). Our novel approach consisted of cutting the frozen tissue into 2–4-mm pieces (<0.04 g) in ice-cold relaxing solution, followed by homogenizing at low speed (1,000 rpm, 3 s) three times (Power Gen 700D; Fisher Scientific) in relaxing solution. The preparation was allowed to settle, and the supernatant was discarded, after which the tissue was resuspended in relaxing solution. The tissue was permeabilized overnight in 1% Triton X-100 (Amresco) at 4 °C, which served to remove cell membranes and intracellular membrane-bound structures. The tissues were then extensively washed in fresh ice-cold relaxing solution, stored on ice, and used within 8 h. The strips of fibers were selected for appearance under a dissecting microscope. They were then attached to a force transducer (KG4A; World Precision Instruments, Sarasota, FL) and high speed length controller (model 315C; Aurora Scientific) using aluminum T-Clips. Muscle dimensions were determined using an ocular micrometer mounted in the dissection microscope (resolution, ∼10 μm). Sarcomere length was measured in the passive relaxed condition by laser diffraction as previously described (21), adjusted to 2 μm and then 2.3 μm. The skinned fibers were incubated with peptide for 5 min in relaxing solution and another 5 min in preactivating solution before measuring the force development. The peptide was also present in all working solutions thereafter. Isometric tension and ATPase activity were measured at various levels of Ca^2+^ activation as previously described (22, 23). Briefly, the isolated muscle was exposed to a range of calcium solutions obtained by proportional mixing of activating and relaxing solutions, and the force generated and ATP consumed were measured simultaneously during the contraction. Activating solution contained 20 mm Ca^2+^-EGTA, 1.55 mm potassium propionate, 6.59 mm MgCl_2_, 100 mm *N,N*-bis(2-hydroxyethyl)taurine; *N,N*-Bis-(2-hydroxyethyl)-2-aminoethanesulfonic acid, 5 mm sodium azide, 1 mm DTT, 10 mm phosphoenolpyruvate, 0.01 mm oligomycin, 0.1 mm PMSF, and 0.02 mm A_2_P_5_, as well as protease inhibitor mixture. Relaxing solution was identical, except it contained 20 mm EGTA, 21.2 mm potassium propionate, and 7.11 mm MgCl_2_. The preactivating solution contained 0.5 mm EGTA, 19.5 mm 1,6-diaminohexane-*N,N,N,N′*-tetraacetic acid, and 21.8 mm potassium propionate. All solutions contained 0.5 mg/ml pyruvate kinase and 0.05 mg/ml lactate dehydrogenase (Sigma) and had an ionic strength of 180 mm, 5 mm free ATP, and 1 mm free magnesium, as determined by the Fabiato program (24).

ATPase activity was measured by a UV-coupled optical absorbance enzyme assay (25). Briefly, ATP hydrolysis into ADP and inorganic phosphate (P_i_) inside the fiber was coupled to the oxidation of NADH to NAD^+^ catalyzed by pyruvate kinase and lactate dehydrogenase. Because NADH absorbs light at 340 nm and NAD^+^ does not, the oxidation of NADH to NAD^+^, and thus ATP consumption, was determined by measuring the absorbance of UV light at 340 nm at steady state within the measurement chamber. This absorbance signal was calibrated by repeated injections of 50 nl of 10 mm ADP into the measuring chamber. The ADP injection induced a rapid step reduction in fluorescence, and the rate of ATP consumption was calculated by the magnitude of this step from the fluorescent decay rate at 340 nm. In addition, the ADP injection served to confirm that the chemical response time and the bath stirring were adequate. Furthermore, the rate of force redevelopment following a release-restretch maneuver, *k*_tr_, was measured during a final contraction at maximum Ca^2+^ as previously described (23). Only muscles that maintained greater than 80% maximal tension were included for analysis.

##### Pulldown Assay

His-tagged recombinant proteins, such as mouse C0-linker, C0-C1f, C0-C2, C0-C3, and C0mC1f, were expressed in *E. coli* and purified using nickel-nitrilotriacetic acid column chromatography at room temperature as described previously (4, 15). Total tissue lysates from normal mouse left ventricular myocardium were prepared in radioimmune precipitation assay buffer (20 mm Tris-HCl, pH 7.5, 150 mm NaCl, 1 mm Na_2_EDTA, 1 mm EGTA, 1% Nonidet P-40, 1% sodium deoxycholate, 2.5 mm sodium pyrophosphate, 1 mm β-glycerophosphate, 1 mm Na_3_VO_4_, and 1 μg/ml leupeptin) at 1 mg protein/ml. 200 μl of lysate was then incubated with 20 μl of nickel-nitrilotriacetic acid-agarose beads (Qiagen) at 4 °C for 2 h and spun down, and the pellet was discarded to remove proteins with nonspecific binding to the beads. The supernatant was then incubated with either His-tagged cMyBP-C protein fragments or SUMO-His-tagged α-TM and 20 μl of beads for 2 h at 4 °C, followed by washing with Tris buffer (50 mm) containing 0.5% Triton X-100, 100 mm NaCl, 10 mm MgCl_2_, 0.1 mm PMSF, and 1× protease inhibitor. Proteins bound to the beads were eluted with Laemmli sample buffer (Bio-Rad) and subjected to Western blot analysis. SDS-PAGE and Western blot analyses were carried out as described previously (2).

##### Statistical Analysis

Tension- and ATPase-pCa relationships were fit with a modified Hill equation (23, 26, 27), and stiffness and tension costs were determined by linear fit to the tension-stiffness and tension-ATPase data, respectively. The data were analyzed using 1) one-way repeated measures analysis of variance for the dose response of control and hC0C1f on maximum force development, 2) an unpaired *t* test for comparing control and hC0C1f, 3) two-way analysis of variance, and 4) a Holm-Sidak post hoc test with a level of statistical significance set at *p* < 0.05. The data are presented as means ± S.E.

## RESULTS

### 

#### 

##### Purification and Characterization of hC0C1f

**FIGURE 1.**
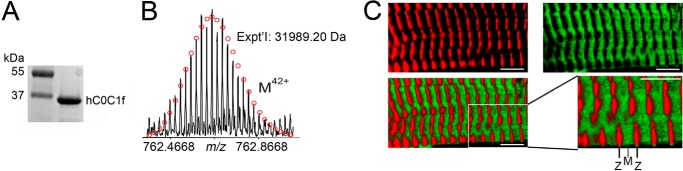
**hC0C1f incorporates into the human cardiac sarcomere.** *A*, recombinant protein was run on SDS-PAGE and SYPRO-RUBY staining to show the purity of recombinant hC0C1f. *B*, electrospray ionization-Fourier transform mass spectrometry spectrum of recombinant hC0C1f. *C*, a skinned stretched cardiomyocyte stained with α-actinin (*red*), a Z-line-specific protein, and incubated with IAF-labeled hC0C1f (*green*). The merged image highlights localization of hC0C1f in the A-band of the sarcomere, and the imaging was performed at SL of 2.1 μm. The *scale bars* represent 5 μm.

**FIGURE 2.**
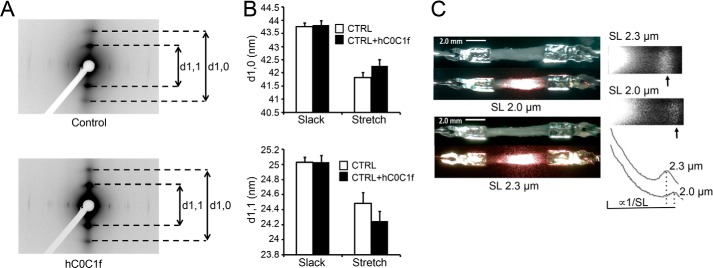
**hC0C1f does not disrupt sarcomere structure.** *A*, representative x-ray diffraction of skinned ventricular fibers with or without hC0C1f. *B*, thin and thick filament spacings indicated by d_1,0_ (thin filament spacing) and d_1,1_ (thick filament spacing) reflections, respectively. *CTRL*, control. *C*, representative laser diffraction pattern and intensity profile of skinned human ventricular muscle tissue at 2- and 2.3-μm SL. Representative images are shown at 5× magnification.

Recombinant hC0C1f was generated, purified, and analyzed with SDS-PAGE showing a pure protein that migrates at ∼40 kDa (Fig. 1*A*). To further characterize the recombinant hC0C1f, high resolution mass spectrometry analysis was performed, confirming the amino acid sequences and a molecular mass of 31,989.20 daltons (Fig. 1*B*). To determine hC0C1f incorporation into the A-band of the sarcomere, permeabilized human ventricular myocytes were incubated with IAF-labeled hC0C1f and fluorescently labeled anti-α-actinin antibodies. IAF-labeled hC0C1f was present specifically between Z-discs, likely on the thin filaments (Fig. 1*C*). This is similar to the localization of endogenous cMyBP-C. To determine whether hC0C1f incorporation disrupted sarcomere structure, x-ray diffraction experiments were performed on permeabilized human donor myocardium at short (2 μm) and long (2.3 μm) sarcomere length (SL) (Fig. 2, *A* and *B*). As expected, spacing between each thin and thick filament was reduced upon stretching. Furthermore, incubation with hC0C1f did not significantly change interfilament spacing at either SL (Fig. 2*B*), suggesting that the presence of hC0C1f in the sarcomere does not adversely affect sarcomere lattice spacing.

##### hC0C1f Disrupts Steady-state Force Development

**FIGURE 3.**
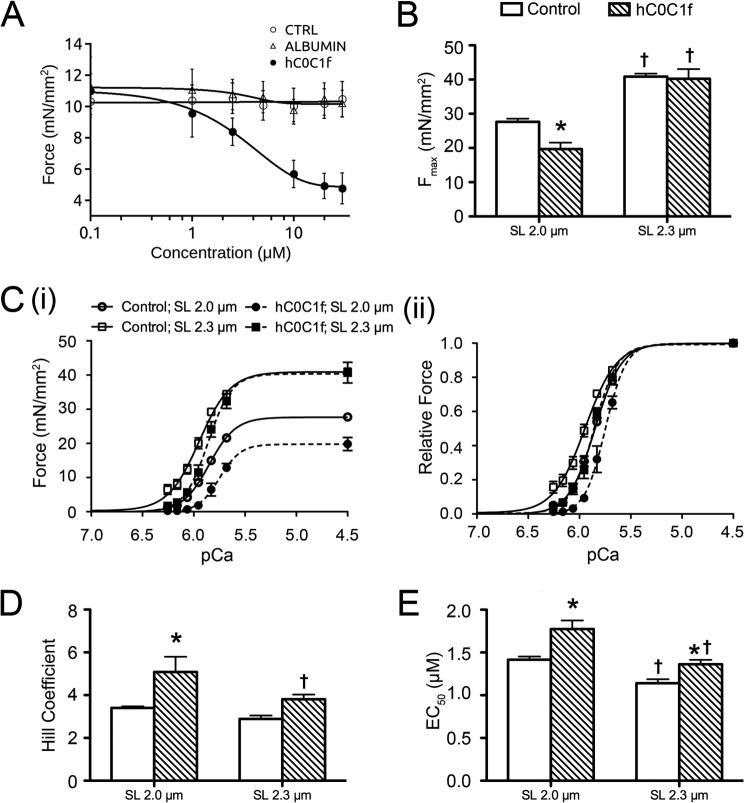
**Altered force-Ca^2+^ relationship and length-dependent activation in the presence of hC0C1f.** *A*, dose-response curve of the inhibitory effect of hC0C1f, compared with vehicle and egg albumin, on force development at pCa_50_ (pCa 5.85) for 2-μm SL. *CTRL*, control. *B*, maximum force (*F*_max_) at pCa 4.5 in skinned ventricular fibers treated with hC0C1f at 2- and 2.3-μm SL. *C*, myofilament force-Ca^2+^ relationship (*panel i*) and force-Ca^2+^ relationship normalized to maximum force (*panel ii*). *D* and *E*, normalized force-Ca^2+^ relationships were fit to a modified Hill equation, and the Hill coefficient (*D*) and EC_50_ (*E*) were determined. *, *p* < 0.05 control *versus* hC0C1f; †, *p* < 0.05 2 *versus* 2.3-μm SL within groups.

To evaluate the effect of hC0C1f on myocardial contractile properties, we developed a novel method to allow for the use of frozen donor heart tissue. The tissue was cut into thin strips to resemble trabeculae and then homogenized, permeabilized, and attached to a length controller and force transducer. The samples were illuminated with a laser to reveal a diffraction pattern allowing SL determination (Fig. 2*C*). To establish a dose-response curve, permeabilized myocardium was incubated at pCa 5.85 (pCa_50_) with increasing concentrations of hC0C1f. As a control, the muscle was also incubated with buffer alone or a control protein, egg albumin, which has a molecular mass similar to that of hC0C1f (∼44 kDa). A dose-dependent reduction in force at pCa_50_ was observed with an EC_50_ (myofilament calcium sensitivity; Ca^2+^ concentration at which half of *F*_max_ is reached) of inhibition at 4.41 μm and maximum inhibition occurring at ∼30 μm of hC0C1f (Fig. 3*A*), with no effect on force development of vehicle or egg albumin. The effects of hC0C1f on tension development and cross-bridge cycling were determined by measuring force-pCa relationships in permeabilized human ventricular myocardium in the absence and presence of 5 μm hC0C1f. Given that length-dependent activation is characterized by an increase in both maximum Ca^2+^-activated force and Ca^2+^ sensitivity upon an increase in sarcomere length, all experiments were done at both short and long SL. This concentration approximates the EC_50_ of inhibition in force induced by hC0C1f. At 2-μm SL, the fragment induced a ∼30% reduction in maximal force (*F*_max_) (Fig. 3, *B* and *C*) and a ∼50% increase in myofilament activation cooperativity, as reported by the Hill coefficient (n_H_) (Fig. 3*D*). Remarkably, hC0C1f did not affect *F*_max_ or nH at long SL. Additionally, hC0C1f induced a rightward shift of the force-pCa relationship at both SLs, indicating decreased myofilament Ca^2+^ sensitivity (∼20% reduction of EC_50_ at both SL) (Fig. 3*E*). Despite decreased myofilament Ca^2+^ sensitivity, length-dependent activation was enhanced with an increased Δ*F*_max_ (difference between maximum force at long and short sarcomere lengths; 13.20 ± 0.41 *versus* 20.56 ± 1.70, control *versus* hC0C1f, *p* < 0.05). Although the ΔEC_50_ (difference between myofilament Ca^2+^ sensitivity at long and short sarcomere lengths) increased, this change was not significant (0.28 ± 0.06 *versus* 0.41 ± 0.07, control *versus* hC0C1f, *p* = 0.07).

##### hC0C1f Alters Cross-bridge Cycling Properties

**FIGURE 4.**
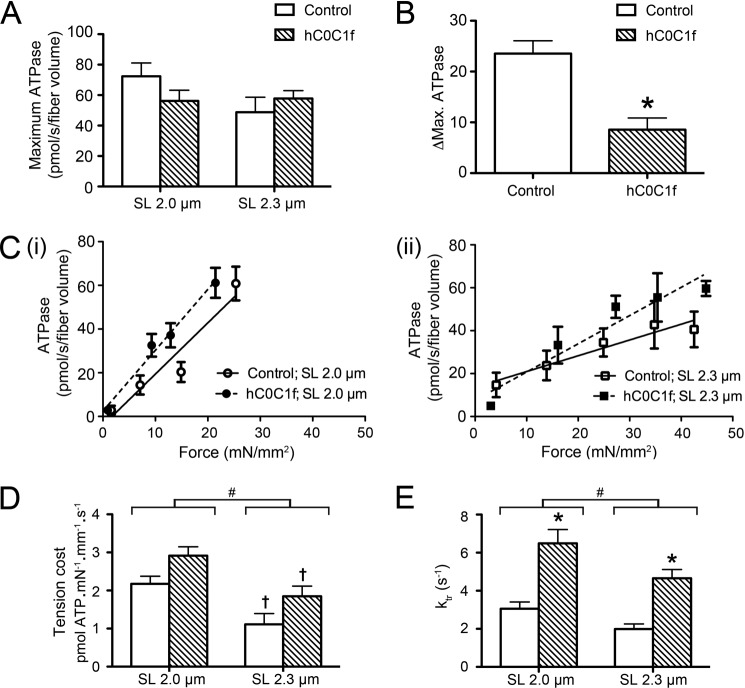
**hC0C1f increases cross-bridge cycling kinetics.** *A*, maximum ATPase hydrolysis rate. *B*, difference in ATPase_max_ at short and long SL. *C*, force-ATPase relationships in various tension ranges (binned data) at 2-μm (*panel i*) and 2.3-μm (*panel ii*) SL, and the force-ATPase relationship of each individual fiber was fit by linear regression. *D*, the slope was then calculated to determine the tension cost. *E*, the rate of force redevelopment (*k*_tr_) was determined following a rapid stretch-release-restretch maneuver. *, *p* < 0.05 control *versus* hC0C1f. #, *p* < 0.05, 2-μm *versus* 2.3-μm SL main effect. †, *p* < 0.05 2-μm *versus* 2.3-μm SL within groups.

We also evaluated myocardium energy consumption by measuring the ATP hydrolysis rate in the absence and presence of 5 μm hC0C1f. Although no significant difference in maximum ATP hydrolysis was observed (Fig. 4*A*), the fragment significantly altered stretch-induced reduction of ATP hydrolysis, as suggested by reduced ΔMax ATPase (Fig. 4*B*). We also observed a significant increase in the tension cost with hC0C1f at both SL (Fig. 4, *C* and *D*). The two-way analysis of variance determined that tension cost decreased with sarcomere length (*p* = 0.0005) and increased with hC0C1f treatment (*p* = 0.0482; main effect). However, hC0C1f did not alter stretch-induced reduction in the tension cost (1.06 ± 0.22 *versus* 1.07 ± 0.26, control *versus* hC0C1f, *p* = 0.9936). The rate of tension redevelopment (*k*_tr_) was also significantly increased in the presence of hC0C1f at both SLs (Fig. 4*E*). These data are consistent with increased tension cost and indicate a significant increase in cross-bridge cycling kinetics induced by hC0C1f.

##### hC0C1f Interacts with the Thin Filament Proteins α-TM and Actin

**FIGURE 5.**
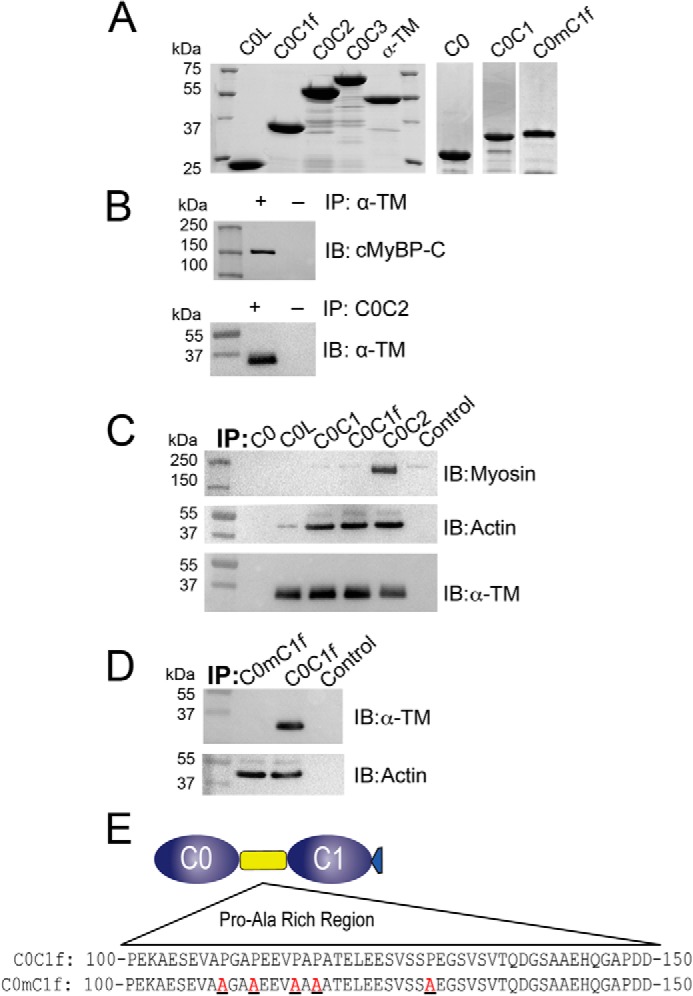
**hC0C1f interacts with the thick and thin filaments.** *A*, SDS-PAGE, followed by Coomassie staining of His tag recombinant proteins containing the N′-terminal domains of cMyBP-C and SUMO-tagged α-TM. *B*, pulldown assay with α-TM (*top panel*) and C0C2 (*bottom panel*), followed by Western blot analysis for cMyBP-C and α-TM, respectively. *C*, pulldown assay with different N′ proteins of cMyBP-C, followed by Western blot analysis for myosin, actin, and α-TM. *D* and *E*, pulldown assay with C0-C1f (*D*) and C0mC1f (with mutated residues in the Pro-Ala-rich region, *E*), followed by Western blot analysis for α-TM and actin. *IB*, immunoblotting; *IP*, immunoprecipitation.

**FIGURE 6.**
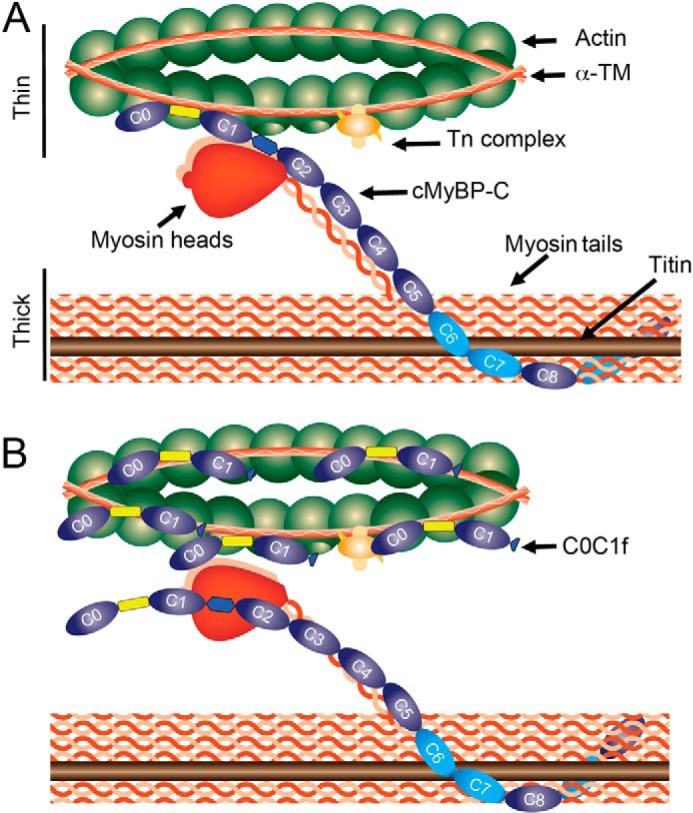
*A*, schematic diagram of the sarcomere with thick (titin, myosin, and cMyBP-C) and thin (α-TM, cTn complex, and actin) filament proteins. cMyBP-C interacts with titin and myosin at its C′-terminal, with actin at the C1 and M domains, myosin at the M domain, and α-TM at the Pro-Ala-linker region. *B*, C0-C1f disrupts native cMyBP-C interaction with the thin filament by binding actin and α-TM at multiple locations. Our data indicate that the presence of C0-C1f fragments in the normal sarcomere disrupts thin filament and thick filament protein interactions, thereby interfering with the regulatory role of native full-length cMyBP-C and, in turn, resulting in diminished force generation.

Previously, we demonstrated that cMyBP-C colocalized with α-TM in the sarcomere (28), and many reports have described its interaction with actin (9, 12). To demonstrate the direct interaction with α-TM and actin, we generated several cMyBP-C N′-terminal recombinant fragments (Fig. 5*A*) and used heart protein lysate to pull down interacting partners. Consistent with previous reports (9), our results show a direct interaction of hC0C1f with actin, but not with myosin (Fig. 5*C*). More interestingly and for the first time, we show a direct interaction of cMyBP-C with α-TM via the Pro-Ala-rich linker region (Fig. 5, *C–E*). This interaction, but not the interaction with actin, is aborted following mutation of five proline residues to alanine (Fig. 5*E*), confirming that the interaction is mediated through the Pro-Ala-rich linker region. These results suggest a direct interaction of cMyBP-C N′-region with both α-TM and actin and that disruption of that interaction by C0-C1f leads to altered cross-bridge cycling. In the present study, we show that incubation with hC0C1f increased cooperativity, cross-bridge cycling, and ATP consumption. These results suggest that hC0C1f fragments constantly interact with thin filament proteins, such as actin and α-TM, thereby altering actomyosin interactions, thereby increasing the probability of strong cross-bridge formation (Fig. 6, *A* and *B*).

## DISCUSSION

Catalytic cleavage of cMyBP-C, in human heart failure and post-MI, has been associated with abnormal contractility; however, the underlying mechanisms have been unclear (2, 3, 29). Previously, we have shown that C0-C1f is released by catalytic cleavage of cMyBP-C at a μ-calpain site and that this release correlated with cardiac insults, such as I/R and MI (2, 30, 31). Further, overexpression of mouse C0C1f resulted in cell death, impaired Ca^2+^ handling, altered contractile function of the sarcomere (4), and, finally, heart failure (3). Using *in vitro* motility and laser trap assays, we also previously determined that C0-C1f is sufficient to reduce actin sliding velocity in a manner similar to that of either C0-C2 or full-length cMyBP-C (13). Furthermore, we determined that the 17 residues of M domain in C0-C1f mediate interaction with actin (4, 13). In this study, we extend these findings to show, for the first time, that the proteolytic fragment hC0C1f can incorporate into the human cardiac sarcomere and that this incorporation is sufficient to 1) decrease maximum force, 2) increase cooperativity, and 3) increase tension cost and cross-bridge cycling. We also show that these effects could be the result of a direct interaction of hC0C1f with both α-TM and actin, inhibiting, in turn, the role of full-length cMyBP-C. This study provides a direct mechanism that explains, at least in part, the depressed contractile function observed post-MI in the noninfarcted myocardium.

Although no attempt has been made to accurately measure the concentration of hC0C1f in the myofilaments following MI or I/R or in heart failure, Western blots done by us and others shown that hC0C1f is easily detectable in myofilaments and is of comparable intensity to the remaining cMyBP-C (2–4). Assuming that cMyBP-C is present at ∼15 μm in cardiomyocytes, we estimate that hC0C1f is present at concentrations ranging from ∼3 to ∼15 μm, depending on the conditions. In the present study, we used a concentration of 5 μm hC0C1f to assay the effects of the fragment on myofilament properties. This concentration is well within the physiological range observed following MI or in heart failure.

Based on the data presented in this study, it is clear that cleavage of cMyBP-C is not the most important/only mechanism leading to contractile dysfunction. Indeed, the human tissue used in these experiments had normal levels of native cMyBP-C, and simple addition of hC0C1f was enough to disrupt the myofilament properties. Of course, proteolysis of cMyBP-C would in itself cause additional dysfunction in the whole organ, and most likely, following I/R or MI, it is the additive effect of less cMyBP-C and the presence of hC0C1f that will lead to contractile dysfunction in the heart. Furthermore, it is still unclear whether the fragment can penetrate neighboring cells. At this time, it is not possible to determine whether the contractile dysfunction seen in the whole heart following I/R or MI is solely due to hC0C1f acting within the cardiomyocyte or due to “diffusion” to remote areas. Along the same lines, it is unclear how long the fragment remains in the cells or attached to the myofilaments. hC0C1f can be detected in tissue several hours to several days following I/R and MI, and we do not know whether there are any mechanisms to actively remove the fragment from the cell. Additionally, it is very difficult to remove hC0C1f from the myofilaments *in vitro* because it does not wash off following removal.

To provide direct support that N′-specific fragments of cMyBP-C are sufficient to incorporate into the sarcomere, Herron *et al.* (7) overexpressed either C0-C1 or C0-C2 regions into neonatal cardiomyocytes and confirmed that these fragments of cMyBP-C could specifically incorporate in the A-band of the sarcomere. However, it remains unclear whether these fragments directly interact with myosin or actin, or both, in a manner that confers stability in the sarcomere. Recent studies demonstrate that cMyBP-C N′-regions structurally reach thin filaments (32, 33) and dynamically interact with actin (12). In the present study, we confirmed that recombinant C0-C1f proteins were able to diffuse into the sarcomere and specifically localize on the A-band and I-band in the presence of endogenous cMyBP-C without changing the interfilament space at both short and longer SL. Interestingly, we did not see any staining of hC0C1f fragments on the Z-line, suggesting that interaction of C0-C1f is very specific to thin and thick filament proteins. However, further studies are needed to define the exact region of interaction that is necessary in the N′-region on the thick and thin filaments. The N′-region of cMyBP-C binds transiently to actin and the myosin S2 region in a phosphorylation-dependent manner to regulate actomyosin interaction and force generation. When the region is cleaved off and C0-C1f is released from the full-length cMyBP-C, transient interaction is lost, and constant interaction occurs. Indeed, we show that the C0-C1f region interacts with actin, but not myosin, because of the absence of M and C2 domains. Furthermore, part of the negative effects of hC0C1f could be due to disruption of the link between the thick and thin filament that native cMyBP-C provides.

In the presence of C0-C2, studies show that maximal force is significantly reduced but that no changes occur in *k*_tr_, tension redevelopment, compared with the untreated control mouse myocytes (7). In contrast, when human myocytes were permeabilized, myocardial preparations were treated with C0-C2, maximal force, and *k*_tr_ values were decreased and increased, respectively, indicating that the difference in *k*_tr_ largely resulted from the presence of β-myosin heavy chain in adult human, compared with adult mouse, ventricle (7). Similarly, in our study, the presence of hC0C1f was able to reduce maximal force and increase *k*_tr_ in human myocytes. It is unlikely that the increase in *k*_tr_ was due to accelerated thin filament activation because this parameter was assessed at maximum saturating levels of activating Ca^2+^, a condition where thin filament activation kinetics is no longer rate-limiting for force development. Interestingly, our study determined that permeabilization of hC0C1f causes a decrease in myofilament Ca^2+^ sensitivity in contrast to the C0-C2 effects. Furthermore, hC0C1f appears to enhance length-dependent activation with increased Δ*F*_max_ and ΔEC_50_. However, this effect is mostly caused by changes in *F*_max_ and EC_50_ at short, but not long, SL, as seen with C0-C2 (7). Therefore, the hC0C1f fragment seems to interfere with thin filament function, as observed by changes in cooperativity and Ca^2+^ sensitivity, at short SL. Conversely, hC0C1f impacts cross-bridge cycling at both SLs, suggesting that it acts on strong cross-bridge kinetics by increasing the off rate. This is supported by the colocalized immunofluorescence images, highlighting the presence of the fragment in both the A- and I-bands.

Consistent with previous studies using various N′ fragments, such as mouse C0-C1 (5, 7), C1-C2 (6), and C0-C2 (7) of cMyBP-C, our results with the physiologically relevant hC0C1f show decreased *F*_max_ and accelerated cross-bridge cycling. Although these studies previously showed increased myofilament Ca^2+^ sensitivity (6, 7), we show decreased EC_50_ at both short and long SLs. This difference might be partly explained by the decreased phosphorylation status of endogenous cMyBP-C seen in failing hearts, compared with the healthy donor tissue used in this study. We and others have previously shown that cMyBP-C phosphorylation is greatly depressed in HF and following MI (2, 34). Moreover, cMyBP-C binding to myosin (31) and actin is modulated by phosphorylation status, and N′-terminal fragments do not bind actin when the cMyBP-C phosphorylation level is high (10, 31). Although Herron *et al.* (7) determined an increase in Ca^2+^ sensitivity following addition of C0-C2 fragment, our report shows that the C0-C1f fragment also contains the first 17 residues of the M-domain, which were sufficient to interact with actin (4, 13). As opposed to the hC0-Clf fragment, C0-C2 preserves the link between thick and thin filaments. Also, C0-C2 has been shown to bind to myosin (10), but hC0C1f does not, suggesting that cMyBP-C interaction with myosin S2 is mediated through M and C2 domains of cMyBP-C.

Recent studies in the literature report that the interaction of cMyBP-C N terminus with thin filaments requires the Pro-Ala-rich region (28, 32, 33, 35, 36). Herron *et al.* (7) proposed that the Pro-Ala-rich region may promote binding of cMyBP-C to thin filament proteins and thus enhance cross-bridge cycling. Here, we show, for the first time, that the hC0C1f region interacts with α-TM via the Pro-Ala-rich region. As suggested by the increased Hill coefficient, hC0C1f interaction with α-TM may alter α-TM movement and indirectly modulate troponin position/structure leading to increased cooperativity. However, more experiments are required to investigate the impact of hC0C1f on α-TM structure and regulation. We postulate that the effects of hC0C1f are primarily mediated by interactions with the thin filament through actin and α-TM. Given the proposed role of cMyBP-C as a brake on actomyosin dynamics (31), we suggest that hC0C1f disables this brake by increasing tension cost and *k*_tr_. This, in turn, mimics the absence of cMyBP-C, increases contraction velocity, and, in the working heart, reduces the systolic phase, similar to what is observed in cMyBP-C knock-out mice (37). This reduced efficiency of contraction then further exacerbates dysfunction in energy-starved hearts.

In conclusion, we show, for the first time, that the cMyBP-C cleavage product hC0C1f is sufficient to cause the contractile dysfunction seen post-MI. We propose that its mechanism of action results from constant interaction with thin filament proteins, such as actin and α-TM. Also, such an interaction disrupts the regulation of actomyosin interaction by affecting endogenous cMyBP-C regulation. Therefore, therapeutic interventions aimed at preventing cleavage of cMyBP-C and attenuating the effects of C0-C1f might confer protection following MI.

## Footnotes

1 The abbreviations used are:

MI: myocardial infarction
cMyBP-C: cardiac myosin-binding protein-C
I/R: ischemia/reperfusion
α-TM: α-tropomyosin
SL: sarcomere length
IAF: 5-iodoacetamidofluorescein.
